# Poly-ADP-ribosylation-mediated degradation of ARTD1 by the NLRP3 inflammasome is a prerequisite for osteoclast maturation

**DOI:** 10.1038/cddis.2016.58

**Published:** 2016-03-24

**Authors:** C Wang, C Qu, Y Alippe, S L Bonar, R Civitelli, Y Abu-Amer, M O Hottiger, G Mbalaviele

**Affiliations:** 1 Division of Bone and Mineral Diseases, Washington University School of Medicine, St. Louis, MO 63110, USA; 2 Department of Orthopaedic Surgery, Washington University School of Medicine, St. Louis, MO 63110, USA; 3 Department of Molecular Mechanisms of Disease, University of Zurich, Zurich, Switzerland

## Abstract

Evidence implicates ARTD1 in cell differentiation, but its role in skeletal metabolism remains unknown. Osteoclasts (OC), the bone-resorbing cells, differentiate from macrophages under the influence of macrophage colony-stimulating factor (M-CSF) and receptor-activator of NF-*κ*B ligand (RANKL). We found that M-CSF induced ADP-ribosyltransferase diphtheria toxin-like 1 (ARTD1) auto-ADP-ribosylation in macrophages, a modification that marked ARTD1 for cleavage, and subsequently, for degradation upon RANKL exposure. We established that ARTD1 proteolysis was NLRP3 inflammasome-dependent, and occurred via the proteasome pathway. Since ARTD1 is cleaved at aspartate^214^, we studied the impact of ARTD1 rendered uncleavable by D214N substitution (ARTD1^D214N^) on skeletal homeostasis. ARTD1^D214N^, unlike wild-type ARTD1, was resistant to cleavage and degradation during osteoclastogenesis. As a result, ARTD1^D214N^ altered histone modification and promoted the abundance of the repressors of osteoclastogenesis by interfering with the expression of B lymphocyte-induced maturation protein 1 (Blimp1), the master regulator of anti-osteoclastogenic transcription factors. Importantly, ARTD1^D214N^-expressing mice exhibited higher bone mass compared with controls, owing to decreased osteoclastogenesis while bone formation was unaffected. Thus, unless it is degraded, ARTD1 represses OC development through transcriptional regulation.

Osteoclasts (OC) are mature myeloid cells, specialized in the removal of aged or damaged bone matrix, which is then replaced by new bone by the osteoblasts.^1^ OC differentiate from precursors of the monocyte/macrophage lineage under the influence of systemic and local factors present in the bone microenvironment.^1^ These factors funnel their inputs through the essential OC regulators, macrophage colony-stimulating factor (M-CSF), which generates mitogenic and survival signals,^2, 3^ and receptor activator of NF-*κ*B ligand (RANKL), whose actions promote OC differentiation and function.^4, 5^ M-CSF and RANKL signaling cascades not only promote the expression of pro-osteoclastogenic transcription factors such as microphthalamia-associated transcription factor (Mitf),^6^ NF-*κ*B,^7^ c-Fos,^8^ NFATc1^9^ and B lymphocyte-induced maturation protein 1 (Blimp1),^10^ but also suppress the expression of the repressors of OC formation, including inhibitors of differentiation,^11^ V-maf avian musculoaponeurotic fibrosarcoma oncogene homolog B (MafB),^12^ interferon regulatory factor-8 (IRF8)^13^ and LIM homeobox 2 (Lhx2).^14^ Thus, OC differentiation is tightly regulated by redundant negative mechanisms to avoid unnecessary osteolysis.

Biochemical signals that regulate OC are amplified and propagated by post-translational protein modifications, mainly phosphorylation,^1, 2^ ubiquitination^15^ and SUMOylation.^16^ Another biochemical modification with potential impact on OC biology is poly-ADP-ribosylation, termed PARylation. This reaction is catalyzed by ADP-ribosyltransferases (ARTDs) also known as poly(ADP-ribose) polymerases (PARPs).^17^ Some ARTDs cause the formation of poly-ADP-ribose (PAR) by transferring several ADP-ribose units from nicotinamide adenine dinucleotide (NAD^+^) onto acceptor proteins. ADP-ribosyltransferase diphtheria toxin-like 1 (ARTD1, also known as PARP1) is the prominent member of this family involved in DNA repair, cell proliferation and survival.^18, 19, 20^ Emerging evidence suggests that ARTD1 also plays an important role in cell fate determination through regulation of transcription.^21^ Indeed, ARTD1 can PARylate transcription factors, thereby affecting their transcriptional activity^22, 23, 24^ or affect gene expression through epigenetic regulation as the negatively charged PAR that it attaches to core and linker histones induce chromatin decondensation, thereby facilitating access of transcription factors to DNA sites.^21^ In addition, ARTD1 can influence chromatin modification by PARylating histones at residues that are also regulated by methylation or acetylation.^21^ Thus, through direct actions on transcription factors and indirect influence through epigenetic mechanisms, ARTD1 may represent a homeostatic mechanism that alters OC differentiation program.

ARTD1 is regulated by various post-translational modifications, including caspase-mediated proteolytic cleavage at aspartate 214,^25^ auto-PARylation,^26^ SUMOylation^27^ and ubiquitination,^28^ which was linked to ARTD1 degradation in cancer cells. Evidence indicates that activation of the NOD-like receptor (NLR) family, pyrin domain-containing 3 (NLRP3) inflammasome, a protein complex comprising the adapter protein ASC and caspase-1, triggers cascade that leads to ARTD1 cleavage.^29, 30^ Despite ARTD1's actions in many tissues, its role in skeletal homeostasis remains unknown as only a few studies have explored its function in osteoclastogenesis *in vitro*.^31, 32, 33^ We found that ARTD1 was degraded during OC differentiation. Conversely, expression of ARTD1^D214N^, which was resistant to degradation, caused a high bone mass phenotype owing to increased expression of OC repressors, decreased OC differentiation and bone resorption, while bone formation was unaltered.

## Results

### ARTD1 is degraded during OC formation through PARylation-dependent mechanisms

**Figure 1:**
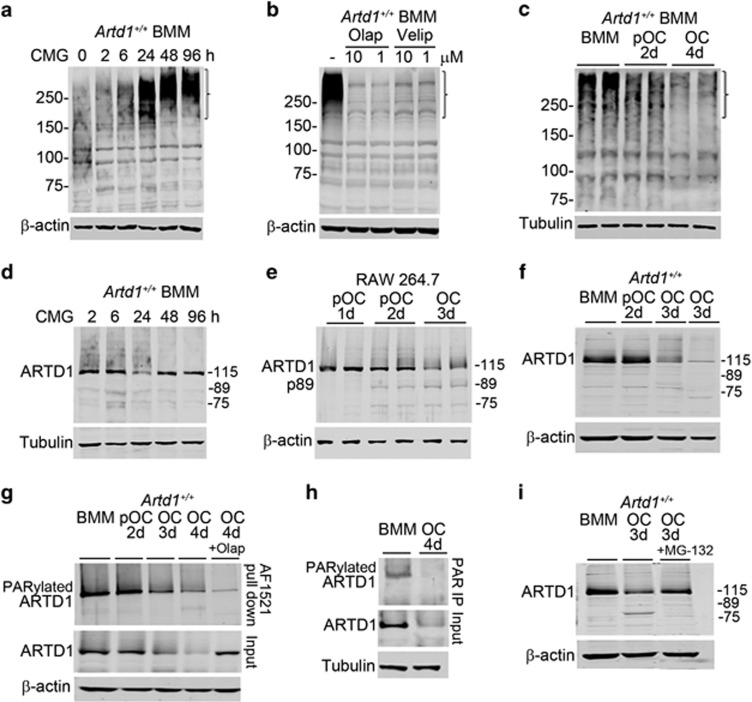
ARTD1 PARylates proteins, including itself, and is degraded during OC formation via the proteasome pathway. (**a**) Western blot analysis using PAR antibody of protein PARylation from 2% CMG-treated BMM cultures; bracket indicates areas of profound changes in PARylation. (**b**) Western blot analysis of the effect of vehicle, olap or velip on 2% CMG-induced protein PARylation for 24 h in BMM cultures using PAR antibody. (**c**) Western blot analysis (using PAR antibody) of protein PARylation during OC differentiation induced by 2% CMG and 100 ng/ml RANKL (experiment was run in duplicates). (**d**) Time-course effect of CMG-containing M-CSF on ARTD1 expression (h, hours of CMG treatment). (**e**) Analysis of ARTD1 during OC differentiation from RAW 264.7 cells. (**f**) Analysis of ARTD1 expression during OC differentiation (d, days of exposure to CMG and RANKL). (**g**) Analysis of ARTD1 during OC differentiation using GST-Af1521 macrodomains. On day 3, cultures were treated with olap, and the experiment was terminated on day 4 (OC, 4 d, +olap). (**h**) Analysis of ARTD1 during OC differentiation by immunoprecipitation using PAR antibody. (**i**) Effect of the proteasome inhibitor, MG-132 on ARTD1 fate. Cultures were treated with 8 μM MG-132 on day 3 for 3 h prior to harvesting samples. Results are from the same gels, but the lanes were cut and pasted. Incubation with MG-132 for >3 h caused cytotoxicity. Data are representative of at least three independent experiments

M-CSF provides growth and survival signals for cells of the OC lineage through regulation of numerous pathways, including PI3K/Akt and MAPK.^1^ Here, we found that M-CSF contained in CMG media^34^ induced massive protein PARylation in mouse bone marrow macrophages (BMM), an effect that was time- (Figure 1a, bracket) and concentration- (Supplementary Figure S1A) dependent, and was inhibited by two chemically different inhibitors of ARTD1 and ARTD2, olaparib (olap) and veliparib (velip) (Figure 1b, bracket) or *Artd1* happloinsufficiency (Supplementary Figure S1B). RANKL decreased protein PARylation in a time-dependent manner (Figure 1c, bracket). Whereas M-CSF treatment did not affect ARTD1 protein levels (Figure 1d, and Supplementary Figures S1B, S1C), RANKL treatment reduced ARTD1 protein abundance (Figure 1e and f). In addition, p89 kDa fragment (p89), a product of caspase-mediated cleavage of ARTD1^30, 35^ was apparent at day 2 during the differentiation of RAW 264.7 cells (Figure 1e), but was not readily detectable in the differentiation of primary BMM (Figure 1f). Thus, ARTD1 is likely responsible for protein PARylation, which inversely correlates with OC differentiation.

Pull-down studies using Af1521 macrodomains (Figure 1g), which have high specificity and affinity for PARylated proteins,^36^ and immunoprecipitation studies using anti-PAR antibody (Figure 1h) showed a decline in the levels of PARylated ARTD1 during OC differentiation. These results suggest that PARylation regulates ARTD1 protein levels. Indeed, in the 3-day OC cultures treated with olap, not only ARTD1 PARylation was inhibited (Figure 1g, top panel), but ARTD1 degradation was also prevented (Figure 1g, middle panel). ARTD1 was degraded through the proteasome pathway since a brief exposure for 3 h of OC cultures to MG-132, a proteasome inhibitor, was able to attenuate ARTD1 loss (Figure 1i). Unexpectedly, p89 remained undetectable even in the presence of MG-132, suggesting slow accumulation of this fragment in these experimental conditions. The protective effect of olap was not due to blockade of OC differentiation because if anything, the number of OC in inhibitor-treated cultures scored at day 4 was higher than that of vehicle-treated cultures counted at day 3 (data not shown). Collectively, these data suggest that ARTD1 auto-modification is required for its degradation during osteoclastogenesis.

### ARTD1 cleavage at D214 is required for its degradation during OC formation

**Figure 2:**
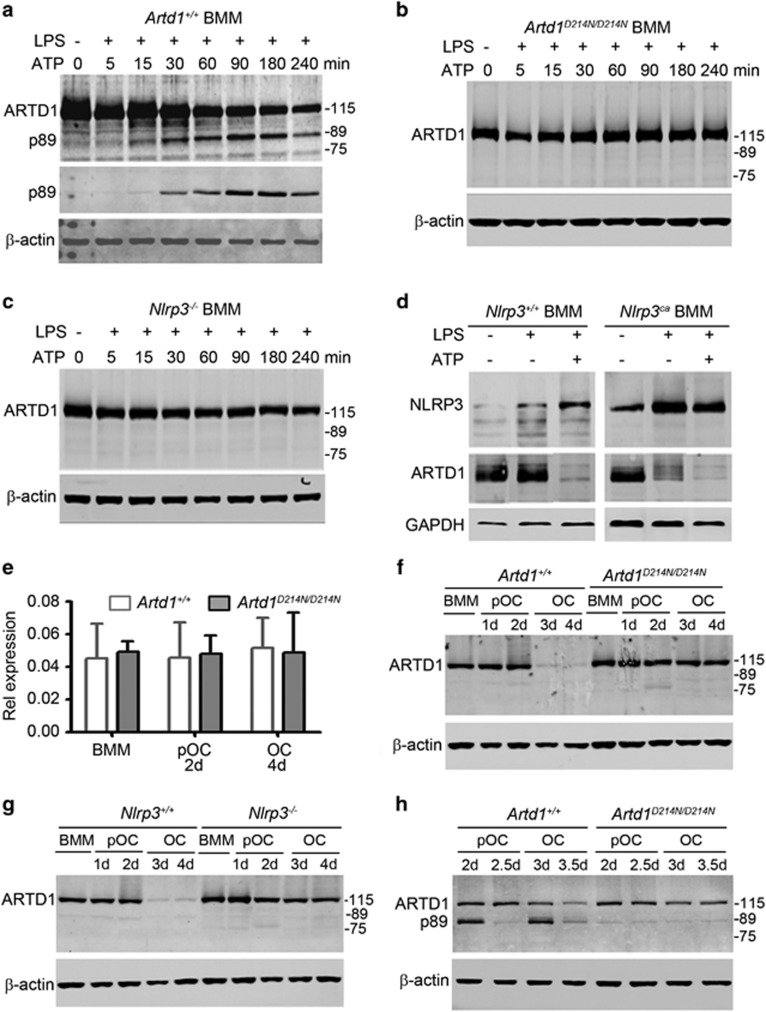
Cleavage of ARTD1 at aspartate 214 is required for its degradation during osteoclastogenesis. BMM isolated from *Artd1*^*+/+*^ mice (**a**), *Artd1*^*D214N/D214N*^ mice (**b**), *Nlrp3*^*−/−*^ mice (**c**), WT mice or mice expressing constitutively activated NLRP3 inflammasome (*Nlrp3*^*ca*^, **d**) were treated with vehicle or 100 ng/ml LPS for 3 h, and exposed to vehicle or 5 mM ATP for the indicated times (min, minutes). Western blot analysis was carried out using ARTD1 antibody (top panel) or p89 ARTD1 antibody (middle panel, **a**). The lanes from the same membranes were cut and pasted in **d**. (**e**) WT and *Artd1*^*D214N/D214N*^ BMM were incubated with 2% CMG (BMM) or 2% CMG and 100 ng/ml RANKL for 2 days (pOC, 2d) or 4 days (OC, 4d), and mRNA expression was analyzed by qPCR. (**f**) Analysis of ARTD1 degradation during OC formation from *Artd1*^*+/+*^ or *Artd1*^*D214N/D214N*^ BMM. (**g**) Analysis of ARTD1 degradation during OC formation from *Nlrp3*^*+/+*^ or *Nlrp3*^*−/−*^ BMM. (**f**) and (**g**) BMM were fed with 2% CMG or 2% CMG and 100 ng/ml RANKL every 2 days. (**h**) Analysis of ARTD1 cleavage and degradation during OC formation from BMM expressing ARTD1^+/+^ or ARTD1^D214N^. BMM were fed with 2% CMG and 100 ng/ml RANKL every day starting at day 1.5, and samples were analyzed 12 h later (day 2 or 3) or 24 h later (day 2.5 or 3.5). Samples were analyzed by Western blot; a nonspecific faint band around 89 kDa can be seen across samples (**f**–**h**). Data are representative of at least two independent experiments

We hypothesized that caspase-mediated cleavage of ARTD1 at aspartate 214 is required for osteoclastogenesis to proceed. Hence, we studied the impact of ARTD1 rendered uncleavable by D214N substitution (ARTD1^D214N^) on skeletal homeostasis. First, to ensure that ARTD1^D214N^ is indeed uncleavable, we exposed LPS-primed BMM to ATP, an activator of the NLRP3 inflammasome.^30^ Inflammasome activation caused a time-dependent decline in ARTD1 abundance in WT BMM, a response that inversely correlated with the levels of the cleaved p89 kDa ARTD1 fragment (p89) (Figure 2a). In contrast, activated inflammasome failed to induce the cleavage of ARTD1^D214N^ (Figure 2b). ARTD1 processing did not occur in *Nlrp3*-deficient mice (Figure 2c) as expected. Conversely, following LPS treatment, which up-regulated NLRP3 as reported,^29^ ARTD1 protein levels were reduced in BMM expressing constitutively activated NLRP3 (NLRP3^ca^) inflammasome in the absence of exogenously added ATP, but not control cells (Figure 2d). Moreover, ARTD1^D214N^ blocked NLRP3^ca^-induced OC formation (Supplementary Figure S2). Thus, ARTD1^D214N^ is resistant to cleavage in response to various stimuli, including pro-apoptotic cues as reported^25^ and NLRP3 inflammasome-induced signals.

We previously reported that the NLRP3 inflammasome is activated during RANKL-induced OC formation in the absence of exogenously added secondary inflammasome-activating signals.^37^ Although ARTD1 is degraded during osteoclastogenesis, it is still unclear whether ARTD1 cleavage at D214 is a prerequisite for its degradation during this process. To understand the relationship between these two non-mutually exclusive mechanisms, we monitored the fate of WT and ARTD1^D214N^ during OC formation. WT and ARTD1^D214N^ mRNA levels were unaltered during OC formation (Figure 2e), and ARTD1 protein levels were diminished by day 3 of cultures in WT cells whereas those of ARTD1^D214N^ protein remained unchanged during OC formation (Figure 2f). Occasionally, a fragment of ~78 kDa was observed in cells expressing ARTD1^D214N^, suggesting that mutant ARTD1 is proteolytically processed to some extent at a different site to enable minimal osteoclastogenesis. Collectively, our results also suggest that WT, but not ARTD1^D214N^ is efficiently degraded during this process. Consistent with a role for the NLRP3 inflammasome in ARTD1 processing, ARTD1 was degraded at a slower pace in NLRP3-deficient cells compared with WT counterparts (Figure 2g).

ARTD1 levels consistently declined by day 3 of OC formation, yet the p89 fragment, which was detectable in RAW 264.7 cells (Figure 1e), remained elusive in primary BMM. Notably, we found that when cells were fed daily (starting at day 1.5) instead of every 2 days as in Figure 2f and g, p89 was readily detected 12 h after media replenishment in WT, but not *Artd1*^*D214N/D214N*^ cells (Figure 2h). These data suggest that NLRP3 inflammasome-mediated ARTD1 cleavage at D214 is the first step in the processing of ARTD1, an event that generates protein fragments which are subjected to full proteolysis during osteoclastogenesis.

### ARTD1 promotes the expression of repressors of OC differentiation through mechanisms involving epigenetic regulation

**Figure 3:**
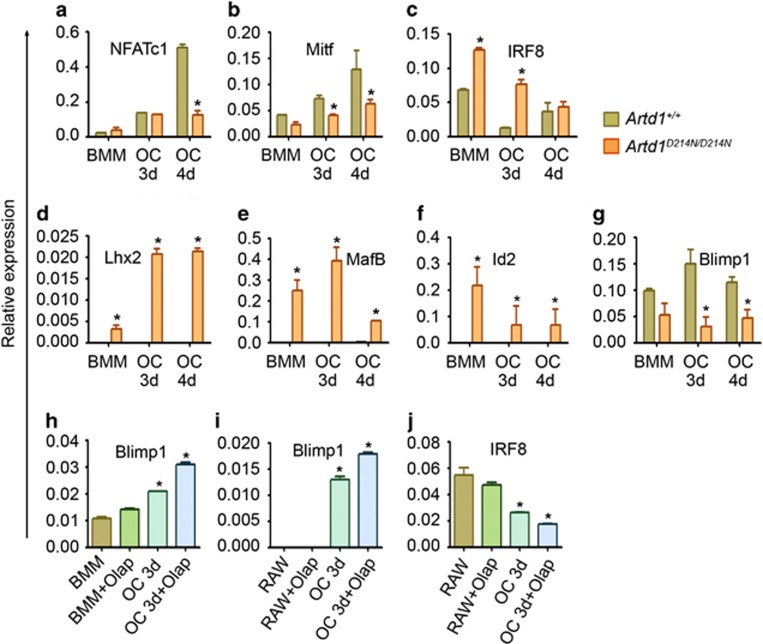
ARTD1 regulates transcription during OC formation. (**a**–**g**) BMM were treated with 2% CMG (BMM) or 2% CMG and 100 ng/ml RANKL for 3 days (OC, 3d) or 4 days (OC, 4d). (**h**) BMM were treated with 2% CMG or 2% CMG and 100 ng/ml RANKL for 3 days in the presence of vehicle or 1 μM olap. (**i** and **j**) RAW 264.7 cells were treated with vehicle or 100 ng/ml RANKL for 3 days in the presence of vehicle or 1 μM olap. RNA were isolated and analyzed by qPCR. Data were normalized to cyclophilin B (relative expression) and expressed as mean±S.D. *P<0.05, *Artd1*^*+/+*^ *versus Artd1*^*D214N/D214N*^ at each time-point (**a**–**g**), cells±olap *versus* BMM (**h**) or cells±olap *versus* RAW 264.7 cells (**i** and **j**)

Given the emerging role of ARTD1 in transcriptional regulation, we analyzed its effects on the expression of transcription factors that regulate OC differentiation. The expression of NFATc1 (Figure 3a) and Mitf (Figure 3b) was up-regulated during the differentiation of WT cells as expected, but not of ARTD1^D214N^-expressing cells. Conversely, expression of the repressors of osteoclastogenesis, IRF8 (Figure 3c), Lhx2 (Figure 3d), MafB (Figure 3e) and Id2 (Figure 3f) declined during the differentiation of WT cells, but was remarkably up-regulated in cells expressing ARTD1^D214N^. Interestingly, the levels of Blimp1 mRNA, a presumed global negative regulator of anti-osteoclastogenic molecules, including IRF8 and MafB^10^ were also lower in *Artd1*^*D214N/D214N*^ BMM (Figure 3g). Whereas ARTD1^D214N^ promoted the expression of the repressors of OC differentiation, inhibition of ARTD1 activity by olap resulted in increased Blimp1 expression (Figure 3h and i) and OC formation (Supplementary Figure S3A) whereas IRF8 expression was decreased (Figure 3j). These results, which are consistent with our recent findings^38^ suggest that ARTD1 activity is an important mechanism in ARTD1 negative regulation of osteoclastogenesis.

**Figure 4:**
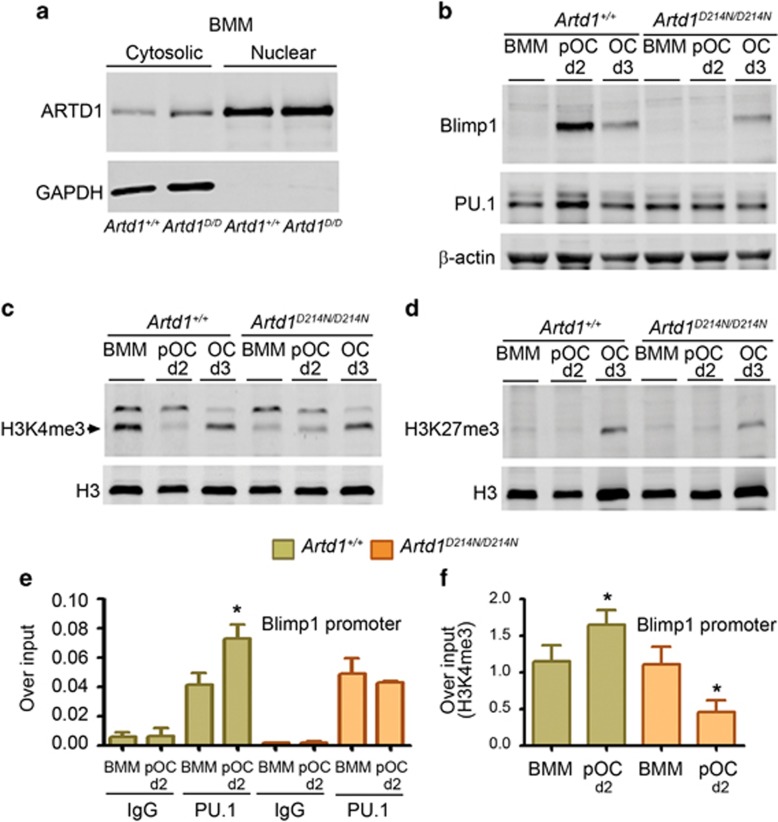
ARTD1 regulates Blimp1 expression through epigenetic mechanisms. (**a**) Western blot analysis of WT ARTD1 and ARTD1^D214N^ expression in BMM *(Artd1*^*D/D*^, *Artd1*^*D214N/D214N*^). (**b**) Western blot analysis of Blimp1 and PU.1 expression in BMM, pOC (d2) or OC (d3). Results are from the same gels, but the lanes were cut and pasted). (**c**) Analysis of global H3K4me3. (**d**) Analysis of global H3K27me3. (**e**) ChIP analysis of PU.1 binding to the Blimp1 promoter using IgG or PU.1 antibody for immunoprecipitation. (**f**) ChIP analysis of H3K4me3 at the Blimp1 promoter. Data are expressed as mean±S.D. *P<0.05, BMM *versus* pOC, and are representative of three independent experiments

We employed chromatin immunoprecipitation (ChIP) to elucidate the mechanisms of ARTD1 regulation of gene expression during osteoclastogenesis. The expression profile of WT and ARTD1^D214N^ was indistinguishable, suggesting that D214N substitution did not affect ARTD1 localization (Figure 4a and Supplementary Figure S3B). Blimp1 protein induction was also attenuated during the differentiation of ARTD1^D214N^-expressing cells compared with WT cells (Figure 4b, top panel) or RAW 264.7 cells (Supplementary Figure S3C); these data were consistent with the profile of Blimp1 mRNA expression (Figure 3g and i).

ARTD1 PARylation of histones can alter the modification of these proteins by methylation or acetylation.^19^ Here, we found that the patterns of global histone3lysine4 trimethylation (H3K4me3), a mark of transcriptionally active chromatin (Figure 4c), but not histone3lysine27 trimethylation (H3K27me3), repressive mark (Figure 4d), was apparently affected by ARTD1^D214N^ expression at day 2 when we never found evidence of ARTD1^D214N^ processing (i.e., generation of 78 or 89 kDa fragment). We therefore focused on the former modification to gain insight onto ARTD1 transcriptional regulation during OC formation. PU.1, the transcription factor that affects the early steps of OC formation,^39^ binds to Blimp1 promoter and positively regulates Blimp1 transcription.^40^ Given the key role of Blimp1 in osteoclastogenesis,^10^ we determined the effect of ARTD1^D214N^ on PU.1 regulation of Blimp1 by focusing on BMM and OC precursors (pOC), which express ARTD1 in contrast to OC. We found that PU.1 expression was increased slightly in WT pOC compared with mutant pOC (Figure 4b, middle panel). Moreover, RANKL-induced PU.1 recruitment to Blimp1 promoter in pOC in WT cells was abolished in ARTD1^D214N^-expressing cells (Figure 4e), consistent with decreased H3K4me3 at the Blimp1 promoter in mutant cells (Figure 4f). Collectively, these results indicate that ARTD1 inhibits OC formation by promoting the expression of the repressors of this process while interfering with the expression of master pro-osteoclastogenic transcription factors such as Blimp1 through mechanisms involving histone methylation.

### Uncleavable ARTD1 causes a high bone mass phenotype in mice

**Figure 5:**
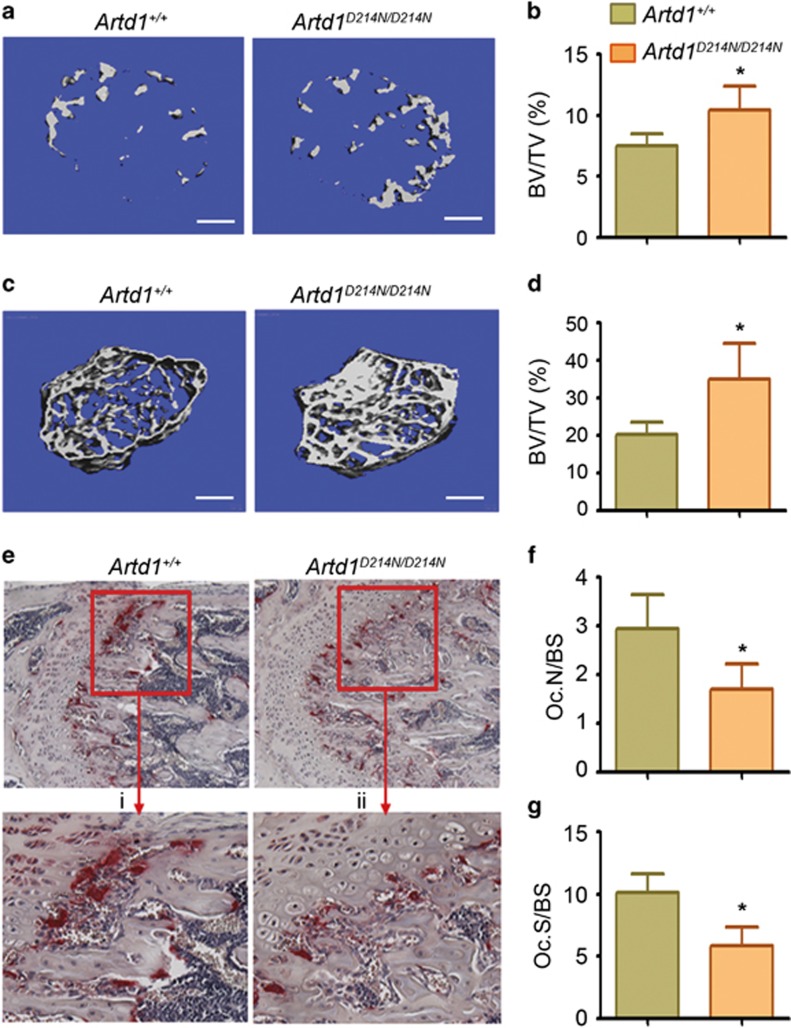
ARTD1^D214N^ causes high bone mass associated with decreased OC number. (**a**) Cross sections of 3 D μCT reconstruction of trabecular bones of distal femoral metaphyses, and (**b**) trabecular bone mass (BV/TV) from 2-week-old *Artd1*^*+/+*^ and *Artd1*^*D214N/D214N*^ male mice. Scale bar, 250 μm. **(c**) Cross sections of 3 D μCT reconstruction of distal femoral metaphyses, and (**d**) BV/TV from 8-week-old *Artd1*^*+/+*^ and *Artd1*^*D214N/D214N*^ male mice. Scale bar, 500 μm. (**e**) TRAP staining of bone sections; (i) and (ii) represent a higher view of the area highlighted by the red box. (**f**) Histomorphometric analysis of OC number/bone surface (Oc. N/BS) from WT and *Artd1*^*D214N/D214N*^ mice. (**g**) OC surface/bone surface (Oc.S/BS). Quantitative data are from 8 *Artd1*^*+/+*^ mice and 5 *Artd1*^*D214N/D214N*^ mice (**b**), and 4 *Artd1*^*+/+*^ mice and 4 *Artd1*^*D214N/D214N*^ mice (**d**, **f** and **g**). Data are expressed as mean±S.D. *P<0.05

To determine the skeletal impact of constitutive expression of ARTD1^D214N^,^25^ we analyzed the femora of mice using micro-computed tomography (μCT). Bone mass was significantly higher in *Artd1*^*D214N/D214N*^ male mice at age 2 weeks (Figure 5a and b) and 8 weeks (Figure 5c and d) of age compared with littermate WT male mice, consistent with higher bone mineral density (BMD), increased number (Tb.N) and thickness (Tb.Th) of the trabeculae, and decreased trabecular space (Tb.Sp) in *Artd1*^*D214N/D214N*^ mice (Supplementary Figure S4A). This high bone mass phenotype was most likely related to defective bone resorption as OC number (Oc.N/BS, Figure 5e and f) and surface (Oc.S/BS, Figure 5g) were decreased in *Artd1*^*D214N/D214N*^ compared with WT mice, consistent with attenuated expression of OC markers, tartrate-resistant acid phosphatase (TRAP) and cathepsin K (Supplementary Figure S4B). In contrast, neither the dynamic indices of bone formation, mineral apposition rate and bone formation rate (Supplementary Figure S5A-D), the number of osteoblasts (Supplementary Figure S5E) nor serum levels of the biomarker of bone formation, procollagen type 1 N-terminal propeptide (Supplementary Figure S5F) were different between the two tested genotypes. Thus, the high bone mass phenotype of *Artd1*^*D214N/D214N*^ mice stems from diminished OC development, but not bone formation.

### Uncleavable ARTD1 causes defective osteoclast formation

**Figure 6:**
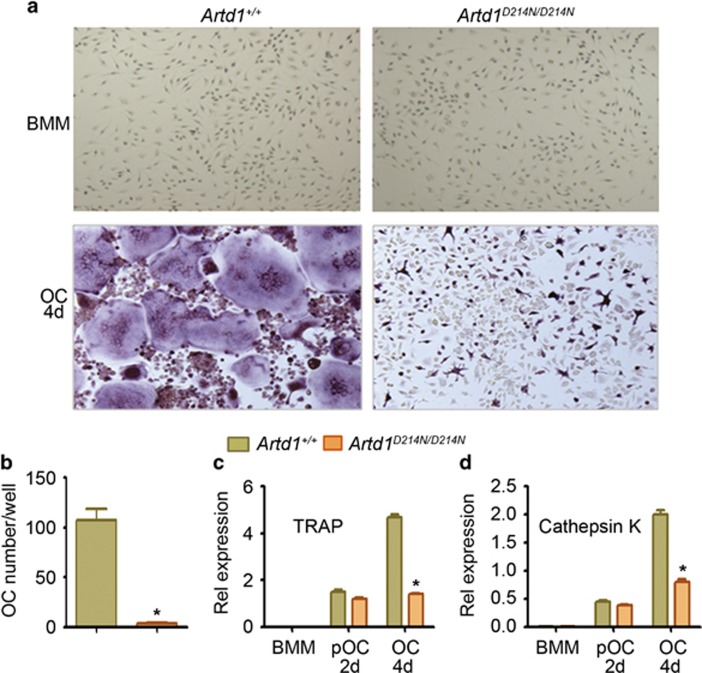
Osteoclastogenesis is defective in cells expressing ARTD1^D214N^. (**a**) WT and *Artd1*^*D214N/D214N*^ BMM were incubated with 2% CMG (top panels) or 2% CMG and 100 ng/ml RANKL (bottom panels) for 4 days (4 d) to generate OC, and stained for TRAP activity. (**b**) OC number. (**c** and **d**) Quantitative PCR analysis of TRAP and cathepsin K mRNA expression after treatment of cells with CMG (BMM) or CMG and RANKL for 2 and 4 days to generate pOC and OC, respectively. Data are representative of at least five (**a** and **b**) or two independent experiments (**c** and **d**), and are expressed as mean±S.D. *P<0.05, *Artd1*^*+/+*^ *versus Artd1*^*D214N/D214N*^ at day 4

The comparable gene expression levels of RANK, osteoprotegerin and RANKL (Supplementary Figure S4B) in bone samples from *Artd1*^*+/+*^ and *Artd1*^*D214N/D214N*^ mice suggests that ARTD1 regulates OC differentiation downstream of RANK signals. Thus, to directly test our hypothesis that the high bone mass phenotype of *Artd1*^*D214N/D214N*^ mice was caused by defective OC development, we treated BMM cultures with M-CSF or M-CSF and RANKL. M-CSF-induced cell expansion was comparable between groups (Figure 6a, top panels). In contrast, OC differentiation was decreased >90% in cultures of ARTD1^D214N^-expressing cells (Figure 6a, bottom panels, and Figure 6b). TRAP (Figure 6c) and cathepsin K (Figure 6d) mRNA expression was also comparable between genotypes at day 2, but was significantly reduced in *Artd1*^*D214N/D214N*^ relative to WT cells by day 4 of cultures. *Artd1*^*D214N/D214N*^ BMM ultimately formed OC when the cultures were maintained for 2 additional days (data not shown), suggesting that OC formation from *Artd1*^*D214N/D214N*^ BMM was attenuated but not blocked, consistent with the reduced number of OC *in vivo* in *Artd1*^*D214N/D214N*^ mice (Figure 5e and f). Thus, ARTD1^D214N^ impairs the intrinsic ability of BMM to efficiently undergo osteoclastogenesis *in vitro*.

## Discussion

The expression of ARTD1 mRNA is maintained during OC differentiation, yet WT ARTD1 protein is barely detectable in OC, suggesting either mRNA translation inhibition by noncoding RNA or ARTD1 protein degradation during OC formation. Although ARTD1 is a target of microRNA such as miR-223,^41^ our results strongly support the latter scenario as ARTD1^D214N^ protein was consistently present in OC, and loss of WT ARTD1 in OC was attenuated upon acute pharmacological blockade of the proteasome pathway. The notion that ARTD1 is degraded in OC implies that this protein functions as an intrinsic inhibitor of OC development, a view that is consistent with the enhanced anti-osteoclastogenic potential of degradation-resistant ARTD1^D214N^, and our other observations indicating that OC formation and bone resorption were enhanced in *Artd1*-deficient mice.^38^ Thus, while ARTD1 is dispensable in non-stress states in certain tissues, it plays a non-redundant cell-context-dependent role in skeletal homeostasis. Although ubiquitination-mediated degradation of ARTD1 in cancer cells was reported,^28^ our findings unravel a novel concept in ARTD1 biology whereby degradation of this protein is a prerequisite for full execution of OC differentiation program.

ARTD1 PARylates itself in pOC in response to M-CSF stimulation, and was subsequently degraded upon RANKL exposure. Although further work is required to explore the link between PARylation and ubiquitination of ARTD1 during OC development, the fact that ARTD1 auto-PARylation occurs early in BMM suggests that this modification may be a switch that targets this protein for proteolysis. Consistent with this concept, inhibition of ARTD1 activity, not only prevented ARTD1 auto-modification, but also stopped its destruction. In addition, a small fraction of WT ARTD1 that escapes degradation is apparently not PARylated (Figure 2f and g, OC 4 d). On the other hand, ARTD1 auto-PARylation followed by cleavage and subsequent release from DNA occurs in response to genotoxic insults,^42^ and is the presumed mechanism that prevents ARTD1 overreaction and excessive consumption of NAD^+^, an important source of cellular energy. Thus, it is reasonable to speculate that promotion of ARTD1 PARylation by osteoclastogenic factors triggers the destruction of this enzyme to preserve NAD^+^ for energy metabolism during osteoclastogenesis.

ARTD1 cleavage into p24 and p89 is a hallmark of apoptosis, though the underlying mechanisms have not been elucidated. Intriguingly, mice expressing ARTD1^D214N^ develop normally^25^ as do mice lacking this protein,^43^ suggesting that ARTD1 cleavage is not essential for cell death. Thus, ARTD1 actions, which include modulation of the function of various transcription factors such as NF-*κ*B^24^ and NFATc1^22, 23^ are more complex than originally thought. Here, we detected p89 during the early steps of osteoclastogenesis when ARTD1 was maximally PAR-conjugated, implying that PARylated ARTD1 may be a high affinity substrate for caspases, including caspase-7, which presumably cleaves ARTD1 in the nucleus in a non-apoptotic manner as proposed previously.^35^ Caspase-7 can be activated by caspase-1, the catalytic component of the NLRP3 inflammasome, a pathway that not only regulates OC differentiation and bone resorption,^37, 44, 45, 46, 47, 48, 49^ but is also critical in ARTD1 proteolytic processing during this process as demonstrated in this study. Consistent with an important role of the NLRP3 inflammasome-ARTD1 axis in the regulation of osteoclastogenesis, pharmacological inhibition or deletion of caspase-1, which attenuates ARTD1 degradation, inhibits OC formation.^37, 38^ Thus, despite the lack of the specific details on the enzyme that cleaves ARTD1 in the OC lineage, our results suggest a sequence of events whereby ARTD1 is highly PARylated in BMM in response to M-CSF, cleaved during RANKL-induced BMM lineage commitment, and finally degraded in late pOC.

**Figure 7:**
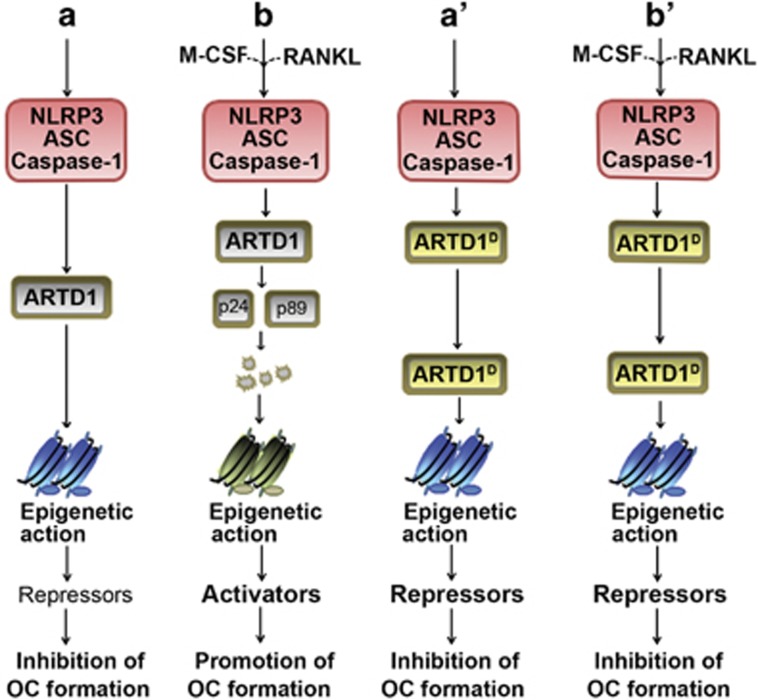
A model of ARTD1 regulation of OC formation. In the absence of M-CSF and RANKL, the NLRP3 inflammasome is minimally active in *Artd1*^*+/+*^ and *Artd1*^*D214N/D214N*^ cells (**a** and **a'**). As a result, the epigenetic action of ARTD1^D214N^ (ARTD1^D^) and WT ARTD1 to a lesser extent, promotes the expression of the repressors of OC differentiation, thereby inhibiting this process. In WT cells, NLRP3 inflammasome activation by M-CSF and RANKL cues leads to ARTD1 auto-PARylation, cleavage and subsequent degradation; this restrains ARTD1-dependent chromatin alterations, and enables the expression of activators and promotion of OC maturation (**b**). In contrast, ARTD1^D^ is resistant to cleavage and degradation (**b'**). Its sustained epigenetic action maintains the expression of OC repressors, thereby, inhibiting OC formation

ARTD1^D214N^ regulates the expression of anti-OC transcription factors (IRF8, Id2, Lhx2 and MafB), but not of pro-OC transcription factors (NFATc1 and Mitf) in BMM. These results suggest that ARTD1^D214N^ mainly regulates the expression of the repressive molecules in BMM, and its inhibitory effects on the expression of late OC markers such as cathepsin K may be an indirect consequence of decreased OC formation. Mechanistically, ARTD1 may regulate transcription in BMM by binding to response elements or secondary hairpin structures of gene regulatory regions. Besides the fact that the role of ARTD1 gene regulation via binding to response elements is still unclear, it is not conceptually obvious to envision that ARTD1 directly regulates the expression of its numerous targets, which are either pro or anti-osteoclastogenic. A plausible alternative is that ARTD1 affects the function or accessibility to DNA response elements^35^ of master transcription factors of OC development through PARylation of these proteins and/or histones. A detailed elucidation of such mechanisms is important, but is outside of the scope of this manuscript. Nonetheless, consistent with this scenario, we found that ARTD1^D214N^ decreased PU.1 binding to the promoter of the master repressor of anti-osteoclastogenic factors, Blimp1,^10^ a response that correlated with H3k4me3. Although PU.1 was not consistently induced in WT pOC (data not shown) it cannot be ruled out that lack of PU.1 induction in ARTD1^D214N^-expressing cells contributed to the attenuated binding of PU.1 to Blimp1 promoter. Collectively, our findings indicate that ARTD1 functions to antagonize OC formation, an effect that is heightened in ARTD1^D214N^, owing to its enhanced stability (Figure 7).

Advanced knowledge on ARTD1 biology revolves around its role in cell survival and death in non-skeletal tissues. We have discovered that ARTD1 impacts bone remodeling through its ability to regulate OC differentiation, hence positioning ARTD1 as an important candidate to regulate bone loss in diseases.

## Materials and Methods

### Mice

Germline knock-in mice globally expressing an ARTD1 mutant rendered uncleavable by D214N substitution (*Artd1*^*D214N/D214N*^ mice) have been previously described.^25^ *Nlrp3*^*−/−*^ and *Artd1*^*−/*^^−^ mice were purchased from the Jackson Laboratory (Bass Harbor, ME, USA), and mice expressing constitutively activated NLRP3 (NLRP3^ca^) inflammasome have also been previously described.^44^ Briefly, *Nlrp3*^*fl(+/D301N)*^ mice were crossed with *LysM-Cre* mice to obtain *Nlrp3*^*ca*^ mice. *Nlrp3*^*fl(+/D301N)*^; *Artd1*^*D214N*^ were mated with LysM-Cre;*Artd1*^*D214N*^ mice to generate *Nlrp3*^*ca*^; *Artd1*^*D214N/D214N*^ mice. All mice were on the C57BL6 background, and mouse genotyping was performed by PCR. All procedures were approved by the Institutional Animal Care and Use Committee of Washington University School of Medicine in St. Louis.

### Bone mass and microstructure

Femoral bone structure was analyzed by μCT system (μCT 40; Scanco Medical AG, Zurich, Switzerland) as described previously.^37^ Briefly, femora from 2-week-old and 8-week-old male mice were stabilized in 2% agarose gel, and μCT scans at 55 kVp were taken along the length of the femur as described previously.^37^ The volume of interest analyzed was located just proximal to the growth plate, spanning a height of 350 μm each for the metaphyseal region.

### Histology and histomorphometry

Mice were labeled twice by injection of calcein (15 mg/kg i.p.; Sigma-Aldrich, St. Louis, MO, USA) 5 and 2 days before euthanasia, which was performed under light anesthesia by exsanguination through dorsal aortic puncture. Blood was collected and the serum stored at −80 °C for later assays. Tissue samples were processed as described previously.^37^ Briefly, long bones were fixed in 10% formalin, decalcified in 14% (w/v) EDTA pH 7.2 for 10–14 days at room temperature (RT), embedded in paraffin, sectioned at 5 μm thickness and mounted on glass slides. Stained sections with H&E or TRAP were used for the analysis of osteoblasts and OC, respectively, as described previously.^37^ For dynamic histomorphometric analysis, bones were fixed in 70% ethanol for 24 h, left undecalcified and embedded in methyl methacrylate. Photographs were taken using nanozoomer (Hamamatsu, Hamamatsu City, Japan).

### OC formation

Bone marrow macrophages (BMM) were obtained by culturing mouse bone marrow cells in culture media containing a 1 : 25 dilution of supernatant from the fibroblastic cell line, CMG 14-12, as a source of M-CSF,^34^ a mitogenic factor for BMM, for ~5 days in a 10-cm dish as described previously.^37^ Nonadherent cells were removed by vigorous washes with PBS, and adherent BMM were detached with trypsin-EDTA, plated at 5–10 × 10^3^/well in a 96-well plate in culture media containing a 1 : 50 dilution of CMG and 100 ng/ml RANKL, a required cytokine for OC differentiation. For OC formation from RAW 264.7 cells, 3 × 10^3^ cells/well in a 96-well plate were treated with 100 ng/ml RANKL. Media with supplements were changed every other day, and maintained at 37 °C in a humidified atmosphere of 5% CO_2_ in air.

### TRAP staining

Cytochemical staining for TRAP was used to identify OC as described previously.^37^ Briefly, cells in a 96 well plate were fixed with 3.7% formaldehyde and 0.1% Triton X-100 for 10 min at RT. The cells were rinsed with water and incubated with the TRAP staining solution (leukocyte acid phosphatase kit, Sigma-Aldrich) at RT for 30 min. Under light microscopy, multinuclear TRAP positive cells with at least 3 nuclei were scored as OC.

### mRNA expression analysis

Total RNA was harvested from cells using RNeasy Plus Mini Kit (Qiagen, Hilden, Germany). Complementary DNA was then synthesized with iScript reverse transcription kit (Bio-Rad, Hercules, CA, USA) and quantified using primers listed in Supplementary Table 1. Gene expression was analyzed by SYBR Green gene expression assay (Applied Biosystems, Waltham, MA, USA).

### Chromatin immunoprecipitation

Chromatin immunoprecipitation (ChIP) was carried out using standard procedures. Briefly, cells were washed, scraped with ice-cold PBS, centrifuged, and the pellets were sonicated to generate ChIP DNA fragments (200–600 bp), which were cross-linked using standard protocols. Samples were incubated with either normal rabbit IgG, H3 antibody (Abcam, Cambridge, UK), H3K4me3 (Millipore, Billerica, MA, USA) or PU.1 antibody (Santa Cruz, Dallas, TX, USA) for overnight at 4 °C under rotation, followed by incubation with protein A/G plus agarose beads for 2–3 h at 4 °C. After several washes, precipitated chromatin complexes were eluted, and uncrosslinked overnight at 65 °C with in buffer containing 5 M NaCl, followed by treatment with RNase A and proteinase K. DNA was extracted with QiaQuick PCR purification kit (Qiagen), and quantified by qPCR using primers listed in Supplementary Table S1.

### Expression and purification of Af1521 macrodomains

Af1521 macrodomains were generated as described previously.^36^ Briefly, BL21 bacteria were transformed with the expression plasmid pGEX containing GST-tagged Af1521, which was purified using Glutathione Sepharose 4B (GE Healthcare). The purity of the fusion protein, resuspended in a buffer containing 50 mM Tris-HCl, pH7.5, 150 mM NaCl and1 mM DTT was monitored by staining the gel with coomassie blue after SDS-PAGE.

### Pull-down, immunoprecipitation and Western blot analyses

Cells were lyzed with RIPA buffer (50 mM Tris, 150 mM NaCl, 1 mM EDTA, 0.5% NaDOAc, 0.1% SDS, 1.0% NP-40), and the proteins were quantified and used for immunoprecipitation and/or Western blot analysis. All lysis buffers were supplemented with phosphatase inhibitors (2 mM NaVO4, 10 mM NaF and 1 mM PMSF) and Complete Protease Inhibitor Cocktail (Roche, Basel, Switzerland). Protein concentrations were determined by the BioRad method. For the pull-down analysis, 800 μg proteins were incubated with 20 μg Af1521, followed by washing the beads four times. For the immunoprecipitation studies, 800 μg proteins were incubated with 10 μg PAR antibody followed by incubation with 60 μl protein A/G-agarose (Santa Cruz Biotechnology) and the pellets were washed four times. Proteins were subjected to SDS-PAGE on 4–12% NuPAGE gels (Invitrogen). Proteins were transferred onto nitrocellulose membranes, and incubated with ARTD1 antibody (1 : 1000, Cell Signaling Technologies, Danvers, MA, USA), NLRP3 antibody (1 : 1000, Adipogen, San Diego, CA, USA), *β*-actin antibody (1 : 50 000, Sigma), HSP90 antibody (1 : 2000, Santa Cruz Biotechnology), tubulin antibody (1 : 1000, Santa Cruz Biotechnology) or GAPDH antibody (1 : 1000, Santa Cruz Biotechnology) for 2 h at room temperature, followed by 1 h incubation with secondary goat anti-mouse IRDye 800 (Rockland, Limerick, PA, USA) or goat anti-rabbit Alexa-Fluor 680 (Molecular Probes, Eugene, OR, USA), respectively. The results were visualized using Li-Cor Odyssey Infrared Imaging System (Li-Cor, Lincoln, NE, USA).

### Statistical analysis

Statistical significance was assessed by Student's *t* test for independent samples, unless otherwise stated.

## Abbreviations

ADP: adenosine diphosphate
ARTD1: ADP-ribosyltransferase diphtheria toxin-like 1
BFR: bone formation rate
Blimp1: B lymphocyte-incuded maturation protein 1
BMM: bone marrow macrophages
BMD: bone mineral density
D214: aspartate 214
Ids: inhibitors of differentiation
IRF8: interferon regulatory factor-8
Lhx2: LIM homeobox 2
MafB: V-maf avian musculoaponeurotic fibrosarcoma oncogene homolog B
MAR: mineral apposition rate
M-CSF: macrophage colony-stimulating factor
μCT: micro-computed tomography
Mitf: microphthalamia-associated transcription factor
NFATc1: nuclear factor of activated T cells, cytoplasmic 1
NAD^+^: nicotinamide adenine dinucleotide
NLRP3: NOD-like receptor (NLR) family, pyrin domain-containing 3
OC: osteoclast
Oc.N/BS: OC number/bone surface
Oc.S/BS: OC surface/bone surface
OPG: osteoprotegerin
PARP1: poly(ADP-ribose) polymerase 1
P1NP: procollagen type 1 N-terminal propeptide
RANKL: receptor activator of NF-*κ*B ligand
RT: room temperature
Tb.N: trabecular number
Tb.Sp: Tb space
Tb.Th: Tb thickness
TRAP: tartrate-resistant acid phosphatase
WT: wild-type
