# Supplementary material for: Poly-ADP-ribosylation-mediated degradation of ARTD1 by the NLRP3 inflammasome is a prerequisite for osteoclast maturation
Source: Cell Death Dis. 2016 Mar 24;7(3):e2153–. doi: 10.1038/cddis.2016.58 (PMC4823959; doi:10.1038/cddis.2016.58)
Supplement: Supplementary Information [file cddis201658x1.pdf]

Figure S1

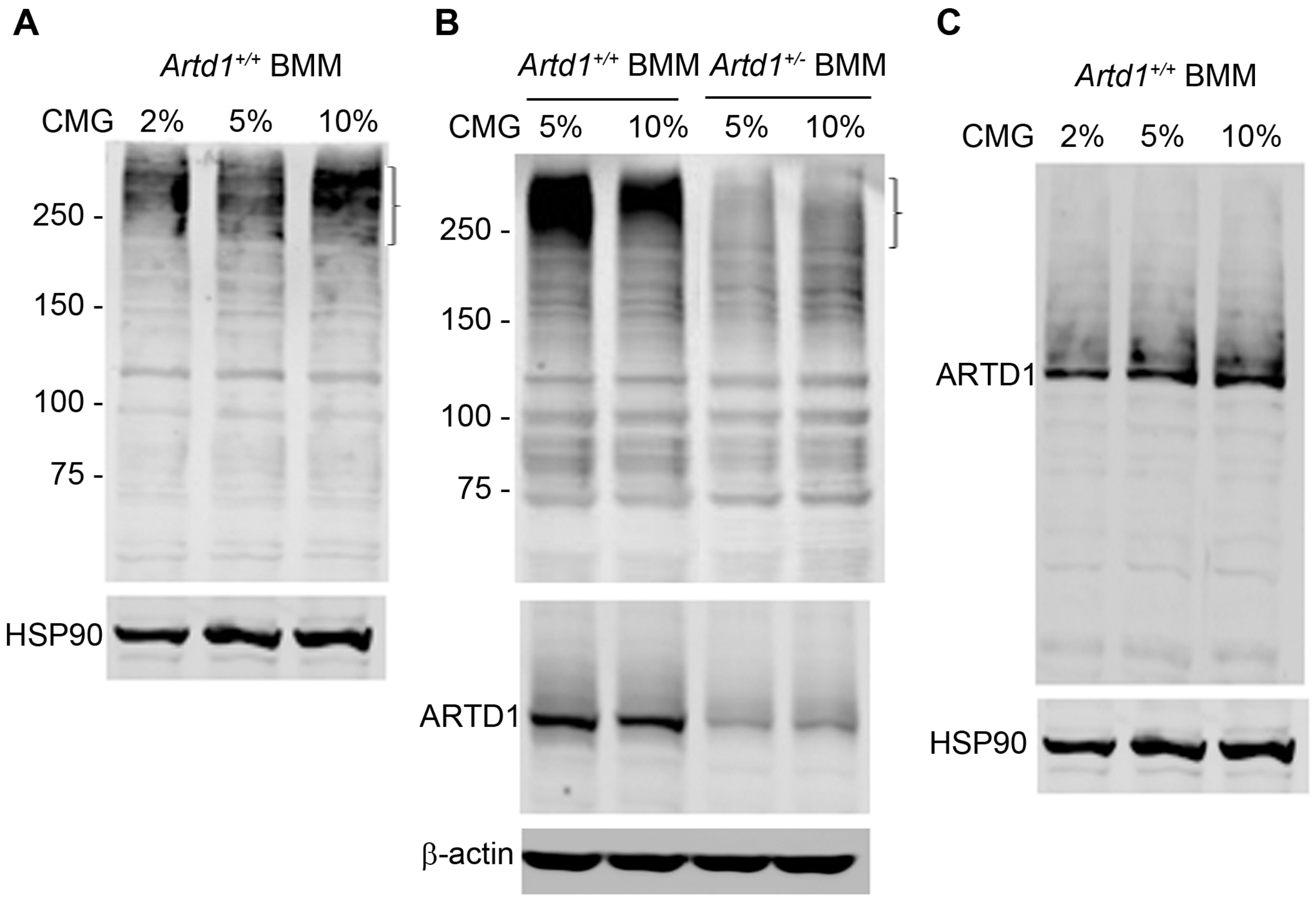
A
Artd1+/+ BMM
CMG 2% 5% 10%
250 -
150 -
100 -
75 -
HSP90
B
Artd1+/+ BMM Artd1+/- BMM
CMG 5% 10% 5% 10%
250 -
150 -
100 -
75 -
ARTD1
β-actin
C
Artd1+/+ BMM
CMG 2% 5% 10%
ARTD1
HSP90

Figure S2

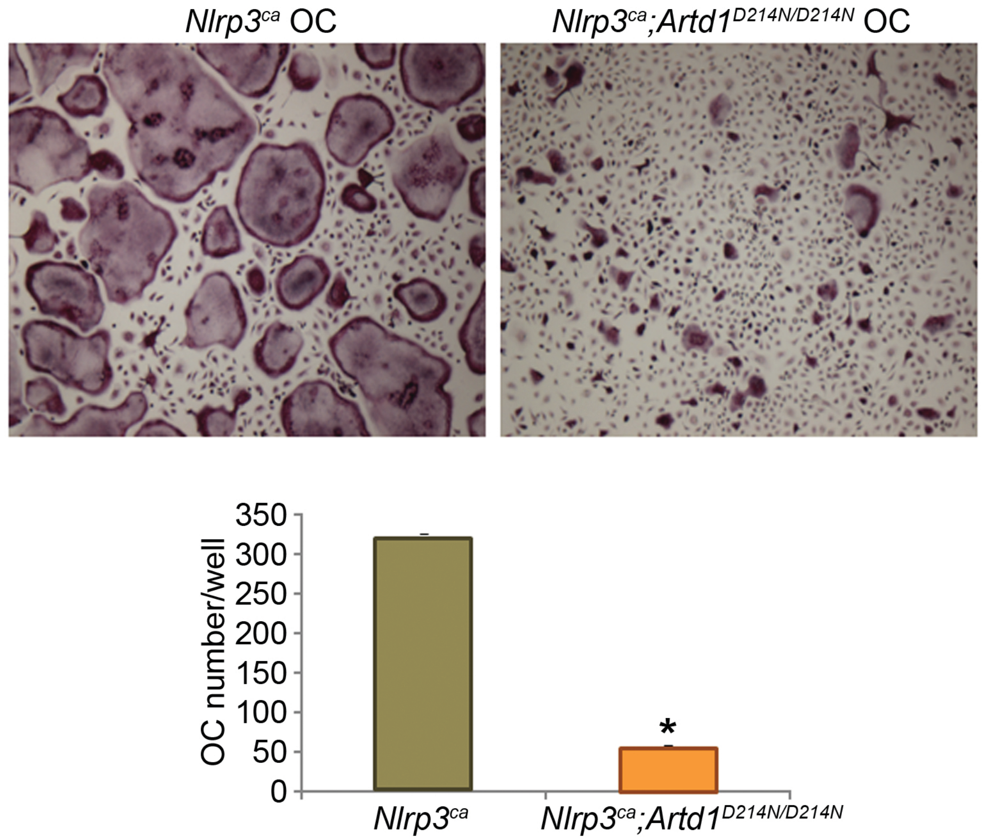

Nlrp3^ca OC
Nlrp3^ca;Artd1^D214N/D214N OC
350
300
250
200
150
100
50
0
OC number/well
*
Nlrp3^ca
Nlrp3^ca;Artd1^D214N/D214N

Figure S3

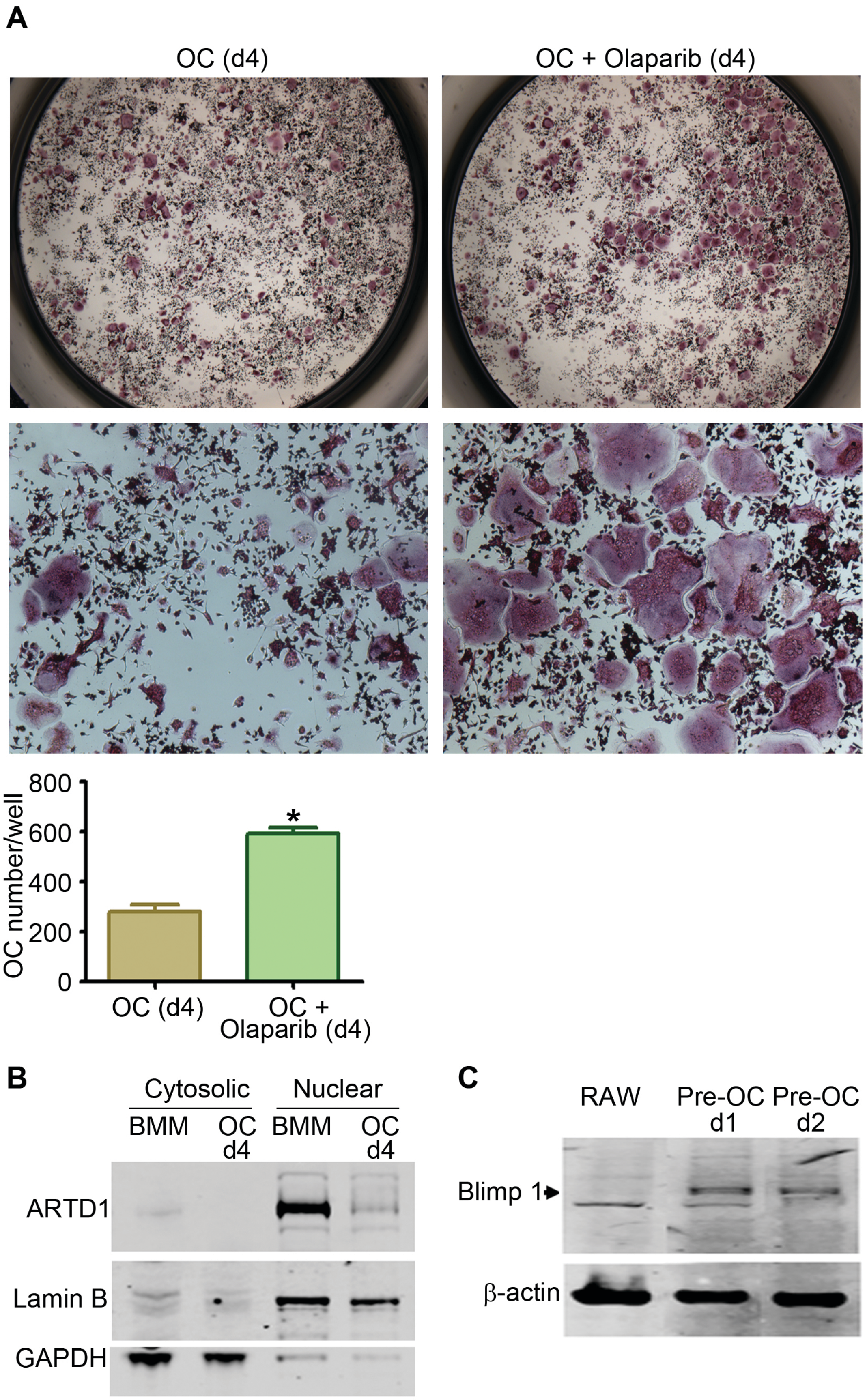
A
OC (d4)
OC + Olaparib (d4)
OC number/well
800
600
400
200
0
*
OC (d4)
OC + Olaparib (d4)
B
Cytosolic
Nuclear
BMM
OC d4
BMM
OC d4
ARTD1
Lamin B
GAPDH
C
RAW
Pre-OC d1
Pre-OC d2
Blimp 1
β-actin

Figure S4

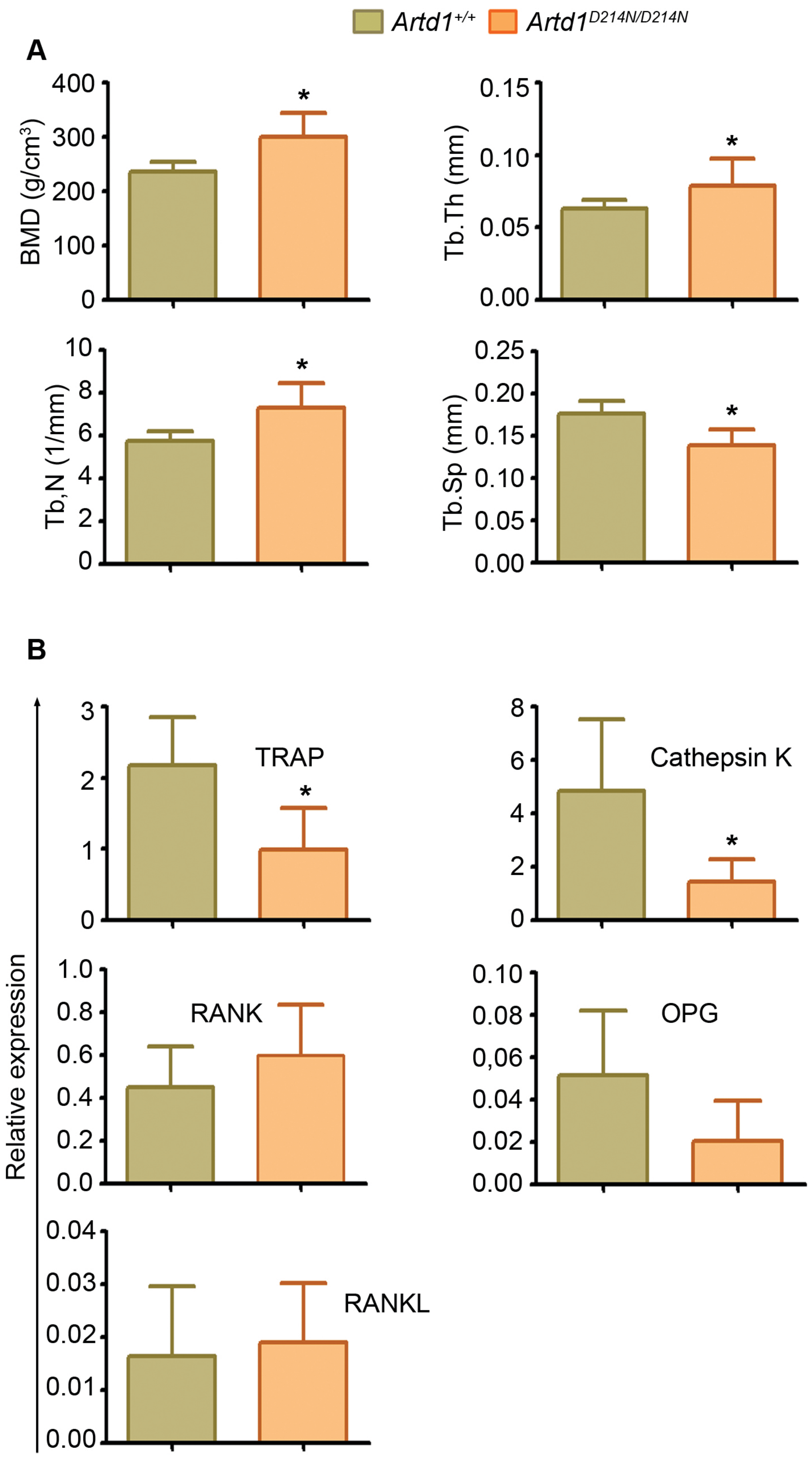

Artd1+/+
Artd1D214N/D214N
A
BMD (g/cm3)
400
300
200
100
0
*
Tb.Th (mm)
0.15
0.10
0.05
0.00
*
Tb,N (1/mm)
10
8
6
4
2
0
*
Tb.Sp (mm)
0.25
0.20
0.15
0.10
0.05
0.00
*
B
Relative expression
TRAP
3
2
1
0
*
Cathepsin K
8
6
4
2
0
*
RANK
1.0
0.8
0.6
0.4
0.2
0.0
OPG
0.10
0.08
0,06
0.04
0.02
0.00
RANKL
0.04
0.03
0.02
0.01
0.00

Figure S5

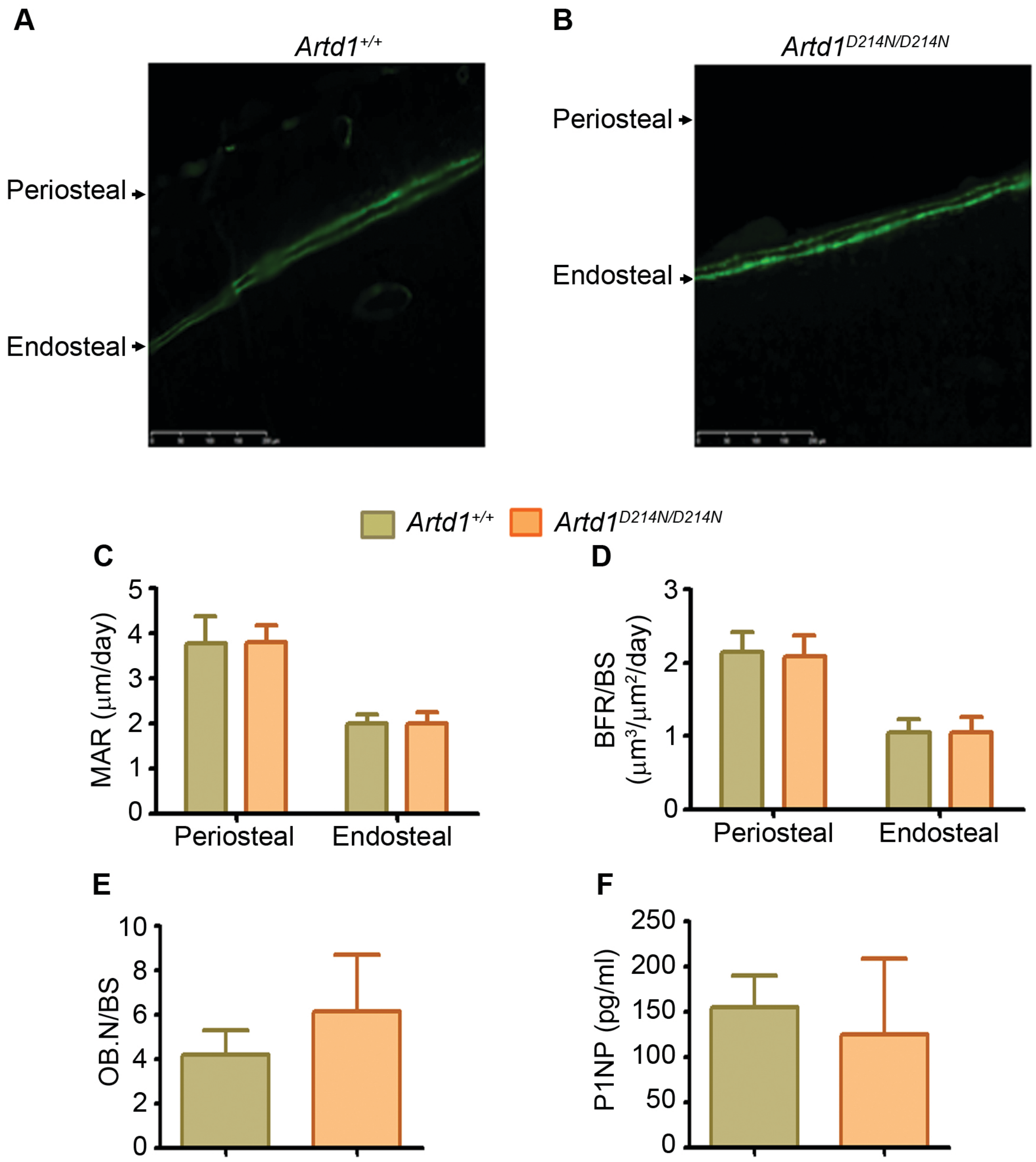
A
Artd1+/+
Periosteal
Endosteal
B
Artd1D214N/D214N
Periosteal
Endosteal
Artd1+/+
Artd1D214N/D214N
C
MAR (μm/day)
0
1
2
3
4
5
Periosteal
Endosteal
D
BFR/BS (μm3/μm2/day)
0
1
2
3
Periosteal
Endosteal
E
OB.N/BS
0
2
4
6
8
10
F
P1NP (pg/ml)
0
50
100
150
200
250

Supplemental Information

Figure S1. CMG-containing M-CSF induces protein PARylation in ARTD1-dependent manner. *Artd1*$^{+/+}$ or *Artd1*$^{+/-}$ BMM were treated with CMG, and samples were subjected to Western blot analysis using PAR {A, B (top panel)}, ARTD1 {(B, middle panel), C}, HSP90 (A, C) or β-actin (B) antibody. The lanes from the same membranes were cut and pasted (B). Bracket indicates areas of profound changes in PARylation.

Figure S2. ARTD1^{D214N} inhibits NLRP3^{ca}-induced OC formation. *Nlrp3*^{ca} or *Artd1*$^{D214N/D214N}$;*Nlrp3*^{ca} BMM were treated with 2% CMG and 100 ng/ml RANKL for 4 days. Cultures were stained for TRAP activity, and OC number was counted. *P < 0.05.

Figure S3. ARTD1 expression inversely correlates with OC formation, and its Inhibition promotes this process. (A) RAW 264.7 cells were treated with100 ng/ml RANKL for 4 days (d, day) in the absence or presence of 1 µM olaparib. Representative pictures from each group run in triplicates were taken at 2X (top panels) or 10X (bottom panels), and OC number was counted. *P < 0.05. (B) BMM were treated with 2% CMG (BMM) or 2% CMG and 100 ng/ml RANKL for 4 days (OC d4). ARTD1 expression was analyzed by immunoblotting. Lamin B and GAPDH were used to monitor the purity of the fractions. (C) RAW 264.7 cells were left untreated (RAW) or treated with 100 ng/ml RANKL for 1 or 2 days, and Blimp1 expression was analyzed by immunoblotting.

Figure S4. ARTD1^{D214N} causes high bone mass, and inhibits the expression of OC markers. (A) µCT analysis of femoral BV/TV of 2-week-old *Artd1*$^{+/+}$ mice (n=8) and *Artd1*$^{D214N/D214N}$ mice (n=5). (B) RNA were isolated bone marrow-free bones from 8 week-old *Artd1*$^{+/+}$ (n=4) and *Artd1*$^{D214N/D214N}$ (n=4) mice, and analyzed by qPCR. Data were normalized to cyclophilin B (relative expression) and expressed as mean ± S.D. *P < 0.05.

Figure S5. Bone formation is unaltered in mice expressing ARTD1^{D214N}. (A) Fluorescence micrographs of calcein labels on the cortical surfaces of bones from 8-week old male mice. (B) Mineral apposition rate (MAR). (C) Bone formation rate (BFR). (D and E) Histomorphometric analysis of osteoblast surface/bone surface (OB.S/BS) and osteoblast number/bone surface (OB.N/BS), respectively, from H&E stained bone sections. (F) Serum levels of P1NP analyzed by ELISA. Quantitative data are from 4 *Artd1*$^{+/+}$ mice and 4 *Artd1*$^{D214N/D214N}$ mice, and are expressed as mean ± S.D.

Table S1. List of primers for qPCR and ChIP.

**Primers**	**Sequence**
Used for qPCR	
NFATC1 Fwd	CCCGTCACATTCTGGTCCAT
NFATC1 Rev	CAAGTAACCGTGTAGCTGCACAA
Cathepsin K Fwd	AGGCAGCTAAATGCAGAGGGTACA
Cathepsin K Rev	AGCTTGCATCGATGGACACAGAGA
Tracp Fwd	CGTCTCTGCACAGATTGCAT
Tracp Rev	AAGCGCAAACGGTAGTAAGG
Cyclophilin B Fwd	AGCATACAGGTCCTGGCATC
Cyclophilin B Rev	TTCACCTTCCCAAAGACCAC
RANK Fwd	GCATCCCTTGCAGCTCAACA
RANK Rev	ATGGAAGAGCTGCAGACCAC
RANKL Fwd	TGTACTTTCGAGCGCAGATG
RANKL Rev	AGGCTTGTTTCATCCTCCTG
PARP1 Fwd	Biorad Unique Assay ID qMmuCID0005676
PARP1 Rev	Biorad Unique Assay ID qMmuCID0005676
OPG Fwd	TCCCGAGGACCACAATGAAC
OPG Rev	TGGGTTGTCCATTCAATGATGT
Mitf Fwd	GTGCAGACCCACCTGGAAAAC
Mitf Rev	AGTTAAGAGTGAGCATAGCCATAG
Lhx2 Fwd	GAAGGGGCGGCCGAGGAAAC
Lhx2 Rev	GCTGGTCACGGTCCAGGTGC
IRF8 Fwd	GAGCGAAGTTCCTGAGATGG
IRF8 Rev	TGGGCTCCTCTTGGTCATAC
MafB Fwd	GAAGCCCGCGAGGCATAT
MafB Rev	GGCCCTGGCACTCACAAA
Blimp1 Fwd	GACGGGGGTACTTCTGTTCA
Blimp1 Rev	GGCATTCTTGGGAACTGTGT
Id-2 Fwd	ATATCAGCATCCTGTCCTTGCAG
Id-2 Rev	GAAATCATGAACACCGCTTATTCAG
18s Fwd	CGGCTACCACATCCAGGAA
18s Rev	GCTGGAATTACCGCGGCT
Used for ChIP	
IRF8 Fwd	CTGCAACGAAAGTCCCTCTC
IRF8 Rev	TTATCGGTTCCCTTGTGTCC
Lhx2 Fwd	AGACCCCCTACTCCAGTTCG
Lhx2 Rev	GGTTCACCCAGGAACAGCTA
MafB Fwd	GCAAGCAAGAAAGCCCTAGA
MafB Rev	GGGAACGAGTCAGGTCGAG
Blimp1 Fwd	GCATGAGAGGCAGGGCAACA
Blimp1 Rev	CCTGCTAACTGAATACATTCAG
